# Biological Properties and Clinical Significance of Lipoprotein-Associated Phospholipase A_2_ in Ischemic Stroke

**DOI:** 10.1155/2022/3328574

**Published:** 2022-10-14

**Authors:** Shuang Zhang, Shuchun Huang, Dingju Hu, Fenglong Jiang, Yanli Lv, Guoqi Liu

**Affiliations:** ^1^Department of Laboratory, Hospital 3201, Hanzhong, 723000 Shaanxi, China; ^2^Department of Neurology, Hospital 302 Attached to Guizhou Aviation Group, Anshun, 561000 Guizhou, China; ^3^Biotecnovo (Beijing) Co. Ltd., Beijing 100176, China

## Abstract

Ischemic stroke, which occurs following blockage of the blood supply to the brain, is a leading cause of death worldwide. Its main cause is atherosclerosis, a disease of the arteries characterized by the deposition of plaques of fatty material on the inner artery walls. Multiple proteins involved in the inflammation response have been identified as diagnosing biomarkers of ischemic stroke. One of these is lipoprotein-associated phospholipase A_2_ (Lp-PLA_2_), an enzyme that can hydrolyze circulating oxidized phospholipids, generating proinflammatory lysophosphatidylcholine and promoting the development of atherosclerosis. In the last two decades, a number of studies have revealed that both the concentration and the activity of Lp-PLA_2_ are independent biomarkers of ischemic stroke. The US Food and Drug Administration (FDA) has approved two tests to determine Lp-PLA_2_ mass and activity for predicting stroke. In this review, we summarize the biological properties of Lp-PLA_2_, the detection sensitivity and limitations of Lp-PLA_2_ measurement, the clinical significance and association of Lp-PLA_2_ in ischemic stroke, and the prospects of therapeutic inhibition of Lp-PLA_2_ as an intervention and treatment.

## 1. Epidemiology of Stroke

**Figure 1:**
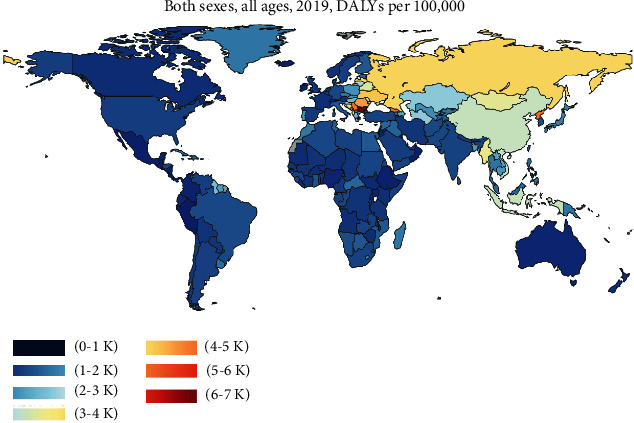
Global prevalence of stroke in 2019. The incidence of stroke per 100,000 people (both sexes and all ages) in different countries. DALYs: disability-adjusted life years. This picture is downloaded from the website of https://www.healthdata.org.

Stroke is a disease caused by brain damage due to the interruption of the blood supply to the brain [1, 2]. Based on data from the Global Burden of Disease Study 2019 (https://www.healthdata.org), stroke is the second leading cause of disability and mortality worldwide (Figure 1). Stroke is normally classified into two subtypes: hemorrhagic stroke and ischemic stroke [3, 4]. A hemorrhagic stroke occurs when a blood vessel ruptures and bleeds into the brain [3, 4]. An ischemic stroke, which accounts for most cases (nearly 87%) [5, 6], is characterized by a sudden loss of blood circulation due to blockage of an artery [5, 6]. Current estimates indicate that stroke affects 13.7 million people and causes more than 5.5 million deaths globally each year [7, 8]. Globally, about a quarter of the population experience an attack of stroke during their lifetimes [7, 8]. In China, the stroke burden has increased over the past 40 years and the prevalence of stroke in Chinese adults aged ≥40 years is 2.58% according to the data from a 7-year (2013-2019) study [9]. Data from another study show that there were more than 3.94 million new stroke cases and 2 million death caused by stoke in China in 2019 [10].

## 2. Pathology and Risk Factors of Ischemic Stroke

**Figure 2:**
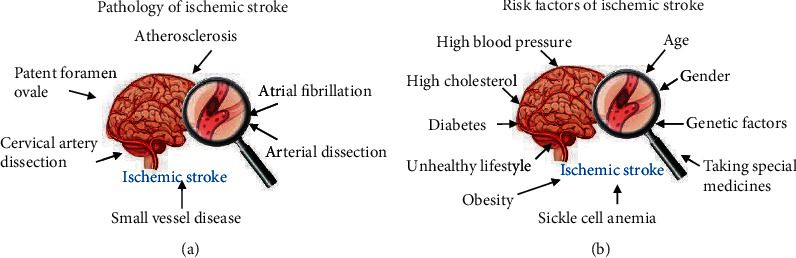
The pathology and risk factors of ischemic stroke. (a) Pathology factors. Different pathology factors, including atherosclerosis, small vessel disease, atrial fibrillation, patent foramen ovale, arterial dissection, and cervical artery dissection, can cause ischemic stroke. (b) Risk factors. Nonmodifiable risk factors (age, gender, and genetic factors) and modifiable risk factors (high blood pressure (≥160/90 mmHg), high cholesterol, diabetes, unhealthy lifestyle, obesity, sickle cell anemia, and taking special) can increase the risk of ischemic stroke.

In most cases, ischemic stroke is thromboembolic, with a common symptom of artery atherosclerosis that leads to fatty deposits and the accumulation of cholesterol plaque in the blood vessels (Figure 2(a)) [11, 12]. Blood clots form in the arteries after the buildup of plaque, and they can block the blood flow to organs, especially the brain, causing ischemic stroke [11, 12]. Small vessel disease is also an important cause of ischemic stroke, especially in Asian populations (Figure 2(a)) [13, 14]. Small vessel disease damages the tiny blood vessels deep inside the brain, reducing blood flow and preventing brain cells from obtaining oxygen and nutrients [13, 14]. Other conditions associated with the pathogenesis of ischemic stroke include atrial fibrillation, patent foramen ovale (PFO), and arterial dissection (Figure 2(a)) [15–17]. Atrial fibrillation is a type of irregular heart rhythm that contributes to thromboembolic stroke through stasis in the left atrium, causing embolization in the brain [15]. PFO is a hole between the two upper chambers of the heart and is present in >25% of the adult population [16]. Blood clots may form in PFO-containing patients when blood flows from one side of the heart to the other and up to the brain [16]. Cervical artery dissection is the most common cause of ischemic stroke in young adults [17]. Arterial dissection occurs after a tear, as blood enters the vessel wall, where it forms a clot and restricts the flow of blood to the brain, causing stroke [17].

The occurrence of ischemic stroke is increased by many risk factors, both nonmodifiable and modifiable. Nonmodifiable risk factors include age, gender, and genetic factors and account for ischemic stroke in nearly 10% of affected patients (Figure 2(b)) [2, 18–21]. Modifiable risk factors include high blood pressure (≥160/90 mmHg), high cholesterol, diabetes, unhealthy lifestyle (e.g., smoking, lacking exercise, and eating unhealthy food), obesity, sickle cell anemia, and the use of controlled drugs (e.g., cocaine and methamphetamines) (Figure 2(b)) [2, 18–21].

## 3. Diagnosis and Biomarkers of Ischemic Stroke

At present, the diagnosis of ischemic stroke relies on both clinical assessment (serum biomarkers) and neuroimaging (computed tomography) [22–24]. Blood tests can identify multiple biomarkers of ischemic stroke, including proteins involved in inflammation responses, such as C-reactive protein (CRP) and matrix metalloproteinases (MMPs), two proinflammatory cytokines (interleukin-6 (IL-6) and tumor necrosis factor-alpha (TNF-*α*)), two cell adhesion proteins (vascular cell adhesion protein 1 (VCAM1) and intercellular adhesion molecule 1 (ICAM1)), and a receptor (N-methyl-d-aspartate (NMDA) receptor) [25–27]. However, these biomarkers are not specific to ischemic stroke and are also used to predict the risks of other inflammatory diseases [25–27]. A panel of four markers, namely, S100 calcium binding protein B (S100B), von Willebrand factor (vWF), MMP9, and VCAM1, can distinguish patients with ischemic stroke from controls with a 90% sensitivity and specificity [25–27]. Another panel of 5 markers, consisting of S100B, vWF, MMP9, B-type neurotrophic growth factor (BNGF), and monocyte chemoattractant protein 1 (MCP1), can distinguish patients with ischemic stroke from healthy controls with 92% sensitivity and 93% specificity [25–27]. Although these biomarkers show promising predictive abilities in distinguishing stroke-risk populations from healthy people, they still present great challenges in distinguishing different inflammatory diseases, such as hemorrhagic stroke, seizure, migraine, syncope, and hypoglycemia [25–27].

## 4. Biological Properties of Lipoprotein-Associated Phospholipase A2 (Lp-PLA_2_)

**Figure 3:**
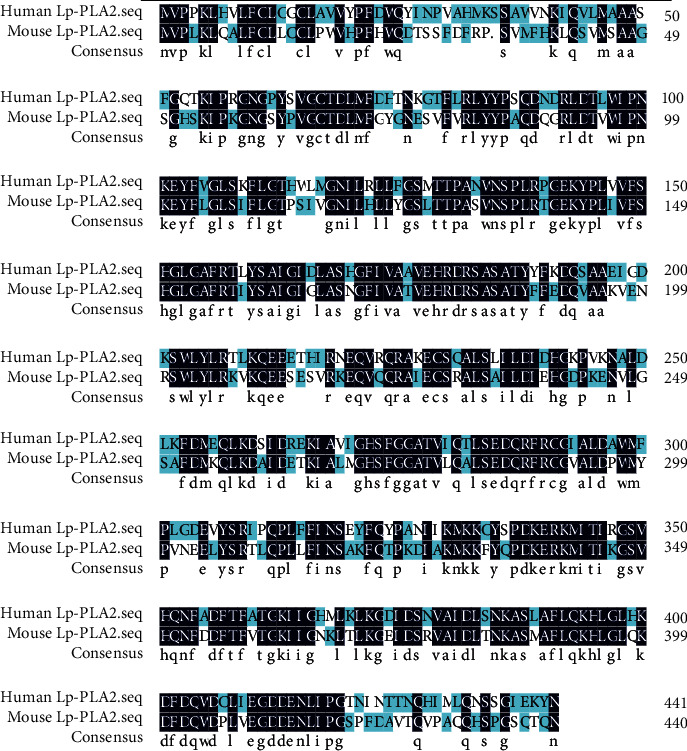
Protein sequence alignment of human Lp-PLA_2_ and mouse Lp-PLA_2_. The amino acid sequences of human Lp-PLA_2_ and mouse Lp-PLA_2_ were subjected for multi alignment using the DNAman software (version 8.0). The same amino acids are shown with dark blue color.

**Figure 4:**
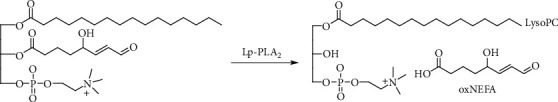
Chemical reaction of Lp-PLA_2_. Lp-PLA_2_ hydrolyzes fatty acids at the sn-2 position of membrane phospholipids, generating free fatty acids and lysoglycerophospholipids.

Mammalian Lp-PLA_2_, also called platelet-activating factor acetylhydrolase (PAF-AH), is encoded by the Phospholipase A2 Group VII gene (PLA2G7) [28–30]. Human Lp-PLA_2_ and mouse Lp-PLA2 share 67.35% amino acid identity (Figure 3). Lp-PLA_2_ belongs to the phospholipase A2 (PLA_2_) family of enzymes, which contains six main subtypes: secreted PLA2 (sPLA_2_), the cytosolic PLA2 (cPLA_2_), calcium-independent PLA2 (iPLA_2_), lysosomal PLA2 (lPLA_2_), Lp-PLA_2_, and adipose-specific PLA_2_ [28–30]. These PLA_2_ enzymes share a common biochemical capability for hydrolysis of fatty acids at the sn-2 position to generate free fatty acids and lysoglycerophospholipids from membrane phospholipids (Figure 4) [28–30].

**Figure 5:**
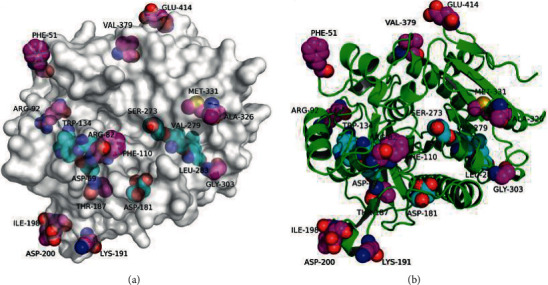
Crystal structure of human Lp-PLA_2_. (a) The molecular surface of human Lp-PLA_2_. (b) A cartoon of the human Lp-PLA_2_ backbone. Pictures are adapted from the reference [31].

Dissection of the crystal structure of Lp-PLA_2_ reveals that the first 17 amino acids constitute a hydrophobic signal peptide (Figure 4) [29, 31–33]. The structure displays an *α*/*β* hydrolase fold with the active site Ser^273^ localized in the Gly-His-Ser-Phe-Gly (GHSFG) motif (Figure 5) [29, 31–33]. This active site consists of a hydrophilic and a hydrophobic region (Figure 5) [29, 31–33]. The hydrophilic region contains a triad composed of Ser^273^, Asp^296^, and His^351^, as well as an oxyanion hole containing Leu^153^ and Phe^274^ (Figure 5) [29, 31–33]. The hydrophobic region contains several residues, namely, Trp^97^, Leu^107^, Phe^110^, Leu^121^, Phe^125^, Phe^156^, Phe^357^, Ile^365^, and Leu^369^, that form pi–pi stacks or undergo van der Waals interactions (Figure 5) [29, 31–33]. Lp-PLA_2_ has an interfacial surface with amphipathic properties that facilitate the binding of its substrates, including PAF, short acyl-chain phosphatidylcholines, oxidized phosphatidylcholines, and phospholipids containing F2-isoprostanes esterified at the *sn*-2 position [29, 31]. The selectivity and specificity of Lp-PLA2 for oxidized and short acyl-chain phospholipid substrates depend on allosteric activation [29, 31].

## 5. Lp-PLA_2_ in the Pathogenesis of Ischemic Stroke

Elevated levels of Lp-PLA_2_ may lead to an increased risk for many diseases, such as coronary heart disease (CHD), myocardial infarction, and ischemic stroke [34, 35]. In the clinical setting, elevated Lp-PLA2 levels have been used as a biomarker to predict the development of CHD in apparently healthy individuals and to predict ischemic stroke [34, 35].

### 5.1. The Secretion and Binding of Lp-PLA_2_

**Figure 6:**
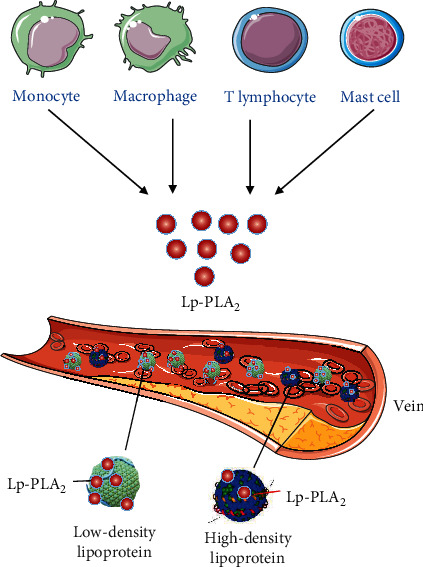
The secretion and binding of Lp-PLA 2. Lp-PLA 2 can be secreted from multiple types of inflammatory cells, such as monocytes, macrophages, T-lymphocytes, and mast cells. In human plasma, Lp-PLA 2 binds to approximately 70% of LDL and nearly 30% of HDL.

Lp-PLA_2_ can be secreted from multiple types of inflammatory cells, such as monocytes, macrophages, T-lymphocytes, and mast cells [29, 36]. In human plasma, Lp-PLA_2_ is estimated to bind to approximately 70% of the low-density lipoproteins (LDL) and nearly 30% of high-density lipoproteins (HDL) (Figure 6) [29, 31]. Lp-PLA_2_ prefers to bind to small, dense, electronegative LDL, and to HDL3c [29, 31].

Other studies have also revealed that low levels of Lp-PLA_2_ are associated with lipoprotein(a) and very low-density lipoproteins. The structure of human Lp-PLA_2_ indicates that residues 114–120 and 360–368 are required for the association with membranes, while residues 192–204 are required for the association with HDL [29, 31].

Unlike its binding in human serum, Lp-PLA_2_ is mostly associated with HDL in the serum of other species, such as the rat, mouse, and pig, where the serum concentrations of LDL are lower than in humans [29]. A structure-based analysis demonstrated that the absence of Trp^115^ and Leu^116^ in the mouse Lp-PLA_2_ may be the cause of varying binding efficiency [37]. The basal levels of Lp-PLA_2_ also differ significantly among species. For example, the basal circulating concentration of Lp-PLA_2_ is nearly 10-fold higher in mouse serum than in human serum [29, 37]. This finding suggests that mimicking human diseases associated with increased Lp-PLA_2_ may be difficult using animal models.

### 5.2. Determination of Lp-PLA_2_ Concentration and Activity

Lp-PLA_2_ has been proposed as a biomarker for the evaluation of the risks of cardiovascular disease (CVD) and ischemic stroke [34, 35]. Multiple methods have been developed to detect both Lp-PLA_2_ concentration (mass) and Lp-PLA_2_ activity in human serum. In 2003, the US Food and Drug Administration (FDA) approved the PLAC test to measure Lp-PLA_2_ concentration using enzyme-linked immunosorbent assays (ELISAs) [38]. The normal range for the Lp-PLA_2_ concentration in serum is less than 200 ng/mL, and people with Lp-PLA_2_ levels of more than 200 ng/mL may be at greater risk of having a stroke [39]. The clinical application of these kits provides doctors with important evidence to diagnose the risks of CVD and ischemic stroke. In December 2014, the FDA approved another PLAC test that could measure Lp-PLA_2_ activity [40]. Comparisons of the association of Lp-PLA_2_ mass and activity in the same materials revealed correlation coefficients ranging from 0.36 to 0.86 and that the accuracy in predicting the risks of CVD and ischemic stroke was greater for Lp-PLA_2_ activity than for Lp-PLA_2_ concentration [41–44]. The risk of CHD and ischemic stroke is approximately 2.1-fold higher in people with PLAC activity over 225 nmol/min/mL than with PLAC activity less than 225 nmol/min/mL [45].

Lp-PLA_2_ can hydrolyze 1-O-hexadecyl-2-acetyl-sn-glycero-3-phosphocholine to 1-O-hexadecyl-2-hydroxy-sn-glycero-3-phosphocholine (LysoPAF) [29]. Lysoplasmalogen-specific phospholipase D (LysoPLD) acts on LysoPAF, and hydrolytically released choline can be detected by choline oxidase [29]. Using this reaction, Yamaura et al. developed a novel enzymatic method for assaying Lp-PLA_2_ activity in serum [46]. This enzymatic Lp-PLA2 activity assay requires only two reagents, thereby enabling a simple two-point linear calibration method with one calibrator. It also does not require inhibitors of esterase-like activity in serum [46].

In 2018, Topbas et al. developed a liquid chromatography–tandem mass spectrometry- (LC-MS/MS-) based assay called “stable isotope standards and capture by antipeptide antibody (SISCAPA) immunoaffinity” to quantify serum Lp-PLA_2_ concentration [47]. The concentrations of serum Lp-PLA_2_ at the peptide level were much higher (up to 8-fold) when determined by the SISCAPA–LC-MS/MS method than by the PLAC test [47]. Importantly, serum Lp-PLA_2_ concentrations measured by SISCAPA–LC-MS/MS show a good correlation with Lp-PLA_2_ activity [47]. The reason for the discordance in serum concentrations of Lp-PLA2 determined by the standard ELISA method versus the SISCAPA–LC-MS/MS method may be due to a blockage of the sensitivity of the ELISA assay due to the interaction between Lp-PLA2 and lipoprotein [47]. The disassociation of Lp-PLA2 from lipoprotein by detergent can significantly increase the Lp-PLA_2_ concentration determined using the PLAC test, while also improving the correlation between Lp-PLA_2_ concentration and activity. Thus, improvement in the ELISA assay by supplementation with detergent may be beneficial for determining Lp-PLA_2_ concentration in the clinic.

Recently, Chen et al. developed a highly sensitive time-resolved fluorescence immunoassay (TRFIA) to detect serum Lp-PLA_2_ concentration in breast cancer patients [48]. TRFIA assay shows better characteristics than ELISA in terms of detection time and measurement range [48]. TRFIA only uses one-step assay and it can be finished within one hour. Moreover, TRFIA shows a 20% higher measurement range than that of ELISA [48].

### 5.3. Clinical Significance of Lp-PLA_2_ in Ischemic Stroke

The results of numerous large-scale observational studies have revealed that Lp-PLA_2_ concentration and activity are both independent biomarkers of ischemic stroke. Most, but not all, observational studies have reported a positive association between Lp-PLA_2_ concentration or activity and ischemic stroke (Table 1).

Persson et al. performed an urban population-based assay in 347 populations (195 patients with CHD and 152 with ischemic stroke) to characterize the association between Lp-PLA_2_ mass or activity and the incidence of CHD and ischemic stroke [49]. After adjusting for age, sex, and cardiovascular factors, the researchers concluded that the elevated Lp-PLA_2_ activity and mass were associated with an increased incidence of ischemic stroke but were not significantly related to CHD occurrence [49]. In the Northern Manhattan Study, Katan and colleagues investigated the association of Lp-PLA_2_ mass and activity with stroke subtypes in a 1,946 stroke-free population [50]. After follow-up for 11 years, 151 participants (7.8%) experienced ischemic stroke at a mean age of 69 ± 10 years [50]. Statistical data revealed no association between either Lp-PLA_2_ mass or activity levels and overall ischemic stroke risk [50]. However, Lp-PLA_2_ mass, but not activity levels, was associated with strokes due to large artery atherosclerosis (LAA), but only among non-Hispanic Whites and not in other racial/ethnic groups [51]. The findings reported by Elkind et al. on the 467 ischemic stroke patients included in the population-based Northern Manhattan Stroke Study indicated that stroke patients with higher Lp-PLA_2_ activity had an increased risk of recurrence after the first ischemic stroke [51]. However, Lp-PLA2 activity was not significantly affected by stroke severity [51].

Cao et al. conducted a study on 200 patients with acute ischemic stroke and 90 healthy controls to investigate the clinical utility of serum Lp-PLA2 activity [34]. They found that the Lp-PLA_2_ concentration was much higher in acute ischemic stroke patients than in the control group and was positively correlated with the National Institutes of Health Stroke Scale score [34]. Lin and colleagues investigated the association between Lp-PLA_2_ level and the early recurrence of vascular events in populations with a transient ischemic attack (TIA) and minor stroke [52]. After adjusting for age and gender, they concluded that Lp-PLA_2_ levels were associated with the incidence of ischemic stroke, myocardial infarction, and death [52]. In a REDUCE-IT (Reduction of Cardiovascular Events With Icosapent Ethyl—Intervention Trial) study, Ridker et al. found that treatment with mineral oil caused a 25% higher risk than Icosapent Ethyl because mineral oil can increase the levels of Lp-PLA_2_, IL-1*β*, IL-6, oxidized LDL, CRP, and lipoprotein(a) [53]. Using a small population (169 patients), Wang reported that plasma Lp-PLA_2_ concentration was associated with acute ischemic stroke in patients with atrial fibrillation [54].

In a systematic review study, Li et al. evaluated the association between Lp-PLA_2_ and CHD in a huge population containing 30,857 participants [55]. The authors found no association between Lp-PLA_2_ activity (or mass) and CHD mortality, whereas they found both Lp-PLA_2_ activity and mass to be independent factors associated with the incidence of cardiovascular events [55]. In another meta-analysis, the Emerging Risk Factors Collaboration found that the Lp-PLA_2_ level was an independent risk factor for cardiovascular events, with a hazard ratio of 1.12, after analyzing the data from 32,075 participants [56]. In 2007, the American Association of Clinical Endocrinologists (AACE) and the American College of Endocrinology (ACE) published guidelines for the management of dyslipidemia and prevention of cardiovascular disease, with the recommendation that patients at risk of cardiovascular or stroke be evaluated for serum Lp-PLA_2_ activity and mass [57]. In a substudy of the Third China National Stroke Registry that included 10,472 participants, Li et al. found that IL-6 and YLK-40 were more apparent than CRP and Lp-PLA_2_ mass and activity to predict recurrent stroke after ischemic stroke [58].

## 6. Inhibition of Lp-PLA_2_

The important roles of Lp-PLA_2_ in the pathogenesis of cardiovascular diseases and ischemic stroke suggest that it may be a potent therapeutic target. In vitro biochemical studies and in vivo animal experiments show that several Lp-PLA_2_ inhibitors can significantly inhibit Lp-PLA_2_ activity and prevent the progression of coronary atherosclerosis.

### 6.1. Darapladib

**Figure 7:**
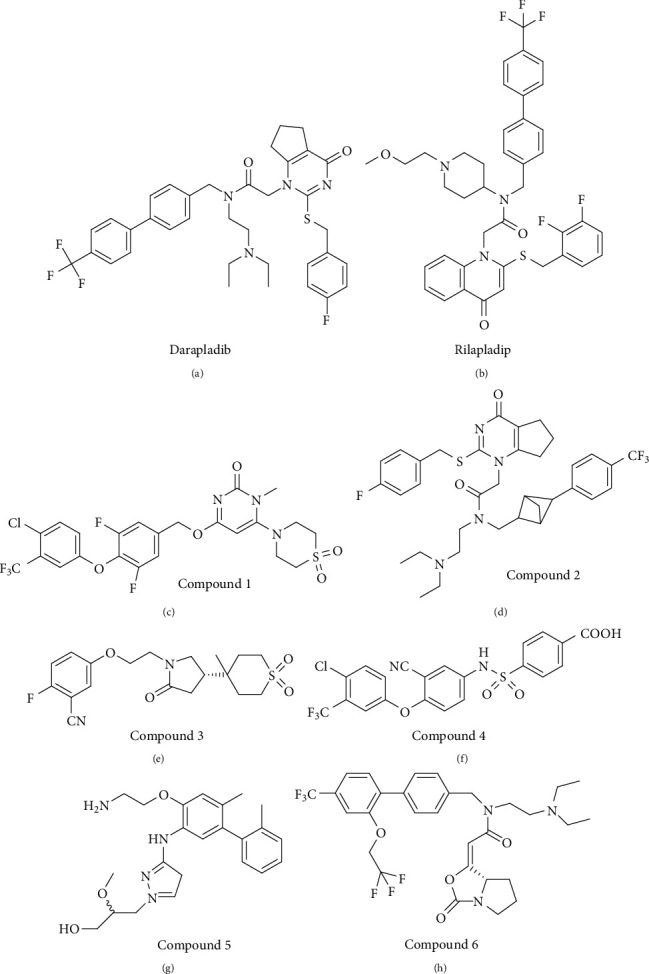
Chemical structures of Lp-PLA_2_ inhibitors. (a) Darapladib. (b) Rilapladib. (C-H) Compound 1-6.

Darapladib (Figure 7(a)), discovered by Human Genome Sciences (HGS) and GlaxoSmithKline (GSK), is a potent inhibitor of Lp-PLA_2_ [59]. In the first two rounds of clinical trials, darapladib showed promising inhibitory effects against Lp-PLA_2_ activity and the inflammatory response [60]. However, GSK announced that darapladib had failed to meet Phase III endpoints in a trial (Stabilization of Atherosclerotic Plaque by Initiation of Darapladib Therapy, STABILITY) with 15,828 CHD patients, because this chemical did not achieve a statistically significant improvement in reducing the occurrence of cardiovascular death, myocardial infarction, and stroke [60]. In another clinical trial (Stabilisation Of pLaques usIng Darapladib–Thrombolysis InMyocardial Infarction 52 (SOLID-TIMI 52)) with 13,026 participants, darapladib (160 mg daily) also failed to reduce the risk of myocardial infarction, urgent coronary revascularization, or death from CHD [61]. The failed clinical trials for darapladib raise concerns regarding the suitability of using Lp-PLA_2_ as a drug target. Instead, it may only be useful as a biomarker for cardiovascular diseases and ischemic stroke.

### 6.2. Rilapladib

Rilapladib (Figure 7(b)) is a small-molecule drug identified from the collaboration between HGS and GSK [62]. A randomized and double-blind clinical trial under phase 2a provided preliminary evidence that the inhibition of Lp-PLA_2_ by administration of 250 mg rilapladib may attenuate the progression of Alzheimer's disease [62]. However, this trial recruited only 124 participants, and the primary outcomes were evaluated at the 24^th^ week [62]. The preliminary findings require repetition in a larger population and for a longer duration. Although rilapladib is an inhibitor of Lp-PLA_2_, animal experiments and human clinical trials are still lacking to confirm the effectiveness of rilapladib in the prevention of ischemic stroke.

### 6.3. Other Compounds

Apart from darapladib and rilapladib, several other compounds also show strong abilities to inhibit Lp-PLA_2_ activity. For example, compound 1 (Figure 7(c)) interacts with the oxyanion hole in Lp-PLA_2_ through two bonds to the backbone amides of Phe^274^ and Leu^153^ [29]. Compound 2 (Figure 7(d)), a bioisostere of darapladib, shows a similar binding mode to that of darapladib, but it has improved physicochemical properties [63]. Huang et al. used a covalent fragment-based approach to identify a highly selective compound 3 (Figure 7(e)), which showed a much higher (130,000-fold) potency and 3,900-fold selectivity compared to a covalent fragment [32]. Three other compounds (4, 5, and 6) (Figures 5(f)–5(h)) also show promising potency for interaction with Lp-PLA_2_ [64–66]. Although in vitro biochemical studies and crystal structure analyses reveal that these compounds significantly inhibit Lp-PLA_2_ activity, these small molecules have not yet been utilized in any human clinical trials. Thus, whether these compounds have potent abilities to improve the outcome of ischemic stroke is unknown.

## 7. Conclusions and Prospects

The global prevalence of ischemic stroke causes mortality and disability in a quarter of the world's population, particularly for people in lower-income and lower-middle-income countries [6]. Stroke can be caused by many risk factors, such as aging, obesity, high blood pressure, alcohol consumption, and lacking physical activity. However, it is still lacking urgent implementation of effective primary prevention strategies at present, suggesting that the stroke burden will continue to increase in the future. To decrease the public health burden caused by stroke, two aspects of work need to be carried out simultaneously. One is to develop reliable stroke biomarkers and efficient detection methods and then screen the general population to predict stoke risk. The second aspect is to develop more effective strategies for stroke treatment in clinical. In response to this, a very important strategy is to conduct in-depth research on the mechanisms of stroke occurrence and development, identify some key therapeutic targets, and develop drugs against these targets.

Evaluation of ischemic stroke risks and treatment are both important in decreasing the global burden [3, 5, 8]. Accumulated evidence has proven that both Lp-PLA_2_ activity and mass are independent biomarkers of ischemic stroke and cardiovascular disease [29]. The US FDA has approved two PLAC assays to measure Lp-PLA_2_ concentration and activity. Some clinical studies have revealed that ischemic stroke shows less association with Lp-PLA_2_ concentration than with Lp-PLA_2_ activity. Thus, the supplementation of detergent in standard PLAC mass assays to disassociate the interactions of Lp-PLA_2_ and lipoprotein may increase the sensitivity and accuracy of ELISA assays. The development of other simple and accurate Lp-PLA_2_ detection methods may also improve the possibility of predicting ischemic stroke risk.

Besides their association with ischemic stroke, elevated Lp-PLA_2_ levels are also common in some other diseases, such as cardiovascular diseases and Alzheimer's disease [29]. Recently, Lp-PLA_2_ is suggested to be a biomarker of severe acute respiratory syndrome coronavirus 2 (SARS-CoV-2) because it is correlated with most of the known comorbidities and complications of this disease [67]. Thus, the ischemic stroke risk cannot be judged solely on the basis of the elevated levels and activities of Lp-PLA_2_. Published studies have paid little attention to the correlations between Lp-PLA_2_ activity (or mass) and other ischemic stroke biomarkers, including CRP, MMPs, IL-6, TNF-*α*, VCAM1, ICAM1, S100B, vWF, BNGF, and MCP1. Panels that combine Lp-PLA_2_ activity (or mass) with these biomarkers may increase the diagnostic accuracy of ischemic stroke risk.

Although different research groups have resolved the crystal structures of Lp-PLA_2_, both alone and when bound with other inhibitors, medicines that can target Lp-PLA_2_ are still lacking for the treatment of ischemic stroke and other diseases associated with the elevations of Lp-PLA_2_ activity or mass. The failure of the clinical trial of darapladib reminds us to reconsider whether Lp-PLA_2_ is truly a suitable target. The observation that *PLA2G7*, the encoding gene for Lp-PLA_2_, is also overexpressed in the pathogenesis of cardiovascular diseases and ischemic stroke suggests that dissection of the upstream regulatory mechanism of *PLA2G7* may benefit the development of therapeutic strategies to decrease Lp-PLA_2_ activity and mass.

## Figures and Tables

**Table 1:** Studies of the clinical significance of Lp-PLA2 in ischemic stroke.

Study year	Sample sizes	Country/region	Ages of participants	Study design	Reported risk factor	Conclusion	References
2018-2020	290	China	50-80 years	Cohort study	Lp-PLA_2_ mass	Lp-PLA_2_ concentration was much higher in acute ischemic stroke patients	[34]
1991-1994	347	Sweden	45–69 years	Cohort study	Lp-PLA_2_ activity and mass	Lp-PLA_2_ activity and mass were associated with the incidence of ischemic stroke	[49]
1993–2001	1946	United States	≥40 years	Cohort study	Lp-PLA_2_ mass	Lp-PLA_2_ mass was associated with the incidence of ischemic stroke	[50]
2009	467	United States	≥40 years	Cohort study	Lp-PLA_2_ activity	Higher Lp-PLA_2_ activity had an increased risk of recurrence in ischemic stroke	[51]
2008-2011	3201	China	≥40 years	Cohort study	Lp-PLA_2_ mass	Lp PLA2 mass was associated with the incidence of ischemic stroke	[52]
2011-2018	8179	United States	Unknown	Cohort study	Lp-PLA_2_ mass, IL-1*β*, IL-6, oxidized LDL, CRP, and lipoprotein(a)	Treatment with mineral oil can increase the levels of Lp-PLA_2_, IL-1*β*, IL-6, oxidized LDL, CRP, and lipoprotein(a) and affect cardiovascular function	[53]
2021	169	China	Average 74.4 years	Cohort study	Lp-PLA_2_ mass	Higher level of Lp-PLA_2_ was a novel biomarker in risk stratification for the incidence of stroke	[54]
2017	30857	China	Unknown	Meta-analysis	Lp-PLA_2_ activity and mass	Lp-PLA_2_ activity and mass were not associated with CHD mortality	[55]
1968-2007	32075	United Kingdom	Unknown	Meta-analysis	Lp-PLA_2_ mass	Lp-PLA2 level was an independent risk factor for cardiovascular events	[56]
2015-2018	10472	China	>18 years	Cohort study	Lp-PLA_2_ activity and mass	IL-6 and YLK-40 were more apparent than Lp-PLA_2_ mass and activity to predict recurrent stroke	[58]

## Data Availability

The data underlying the present study are available on request (corresponding author).
